# Advancing the Use of Restorative Practices to Lessen Inequities in Punitive Discipline and Build Safe, Inclusive, and Nurturing Learning Environments for Traumatized Students

**DOI:** 10.3390/bs16060968

**Published:** 2026-06-11

**Authors:** Corrine Hays, Ylisse Yepez, Hurley Riley, Todd I. Herrenkohl

**Affiliations:** 1 School of Social Work, University of Michigan, Ann Arbor, MI 48109, USA; cghays@umich.edu (C.H.); tih@umich.edu (T.I.H.); 2 Blissfield Community Schools, Blissfield, MI 49228, USA; 3 School of Public Health, University of Michigan, Ann Arbor, MI 48109, USA; rileyhur@umich.edu

**Keywords:** adverse childhood experiences (ACEs), trauma-informed education, restorative practices, exclusionary discipline, school-to-prison pipeline, relationship-building

## Abstract

Childhood trauma, encompassing adverse childhood experiences (ACEs) and racially motivated discrimination, poses significant threats to students’ neurological, social–emotional, and academic development. In school contexts, the behavioral effects are often misinterpreted as willful misconduct and addressed through exclusionary disciplinary measures, perpetuating systemic inequities that disproportionately affect students of color and deepen the school-to-prison pipeline. This article synthesizes research on the intersection between trauma, student learning, and school discipline, emphasizing how trauma-related behaviors are frequently met with responses that fail to address underlying needs. We explore the Trauma-Informed Programs and Practices for Schools (TIPPS) framework as a systems-level model for creating safe, inclusive learning environments. Within this framework, restorative practices are highlighted as a key strategy for reducing reliance on punitive discipline and promoting accountability, relationship-building, and a sense of community. We conclude with actionable recommendations for school practitioners who desire a more active role in restorative practices and advancing trauma-informed, equity-driven system-change consistent with the TIPPS model.

## 1. Introduction

Adverse childhood experiences (ACEs) are defined as potentially traumatic events that can have long-term effects on a child’s development (Felitti et al., 1998). While the primary focus of this article is childhood trauma and trauma-informed school responses, the concept of ACEs is introduced to contextualize the various forms of adversity students may experience. ACEs and trauma, though related, are conceptually distinct: ACEs refer to adverse experiences, whereas trauma refers to a child’s emotional, psychological, and physiological response to adversity (Griffin, 2018). Certain ACEs, such as abuse, neglect, exposure to violence, and discrimination, are traumatic and may affect students’ learning, behavior, and relationships at school (Perfect et al., 2016). Although schools are intended to be spaces for student learning and growth, punitive and exclusionary disciplinary practices may compound the effects of trauma and perpetuate inequities, particularly for students of color and other marginalized youth (Holt et al., 2022; Skiba et al., 2014). The use of restorative practices offers a way to disrupt this pattern and provide opportunities for students who have experienced trauma to learn important skills in conflict resolution. For these reasons, restorative practices are presented as a foundational element of a comprehensive school-based trauma intervention, as elaborated on throughout this article.

## 2. Adverse Childhood Experiences (ACEs)

Children exposed to ACEs may experience significant stress or distress. While ACEs can be traumatic, trauma is defined not by the event itself, but by an individual’s emotional, psychological, or physiological response to the experience (Griffin, 2018). As a result, some children exposed to ACEs may experience trauma-related effects, whereas others may not. ACEs may include exposure to violence, abuse, or neglect; witnessing violence at home or in the community; growing up in a household with substance use or mental health problems; and experiencing parental divorce, economic hardship, and discrimination (Centers for Disease Control and Prevention, 2024; Felitti et al., 1998).

Research suggests that exposure to ACEs is common among school-aged children (Griffin, 2018). According to the National Child Traumatic Stress Network, by age 16, nearly two-thirds of all children in the United States will have experienced at least one ACE (Griffin, 2018). Furthermore, a 2023 study evaluating the prevalence of ACEs in high school students found that more than 75% had experienced one or more ACEs and around 20% had experienced four or more ACEs, suggesting an elevated risk of learning deficits, behavioral and mental health problems, and long-term health challenges (Swedo et al., 2024). Among these individuals, over 60% experienced emotional abuse, while others were exposed to physical abuse (31.8%) and household mental health challenges (28.4%) (Swedo et al., 2024). Notably, in this study, students who identified as female, American Indian or Alaska Native, multiracial, or LGBTQ+ experienced the highest prevalence of ACEs (Swedo et al., 2024).

In the United States, racial minority youth are more likely to experience ACEs, as well as racial discrimination, which can also be traumatic (Kurani et al., 2022). Racial discrimination is the unfair treatment of minoritized students, including harassment, social exclusion, and prejudicial treatment by school staff. This treatment can include being held to different standards on academic tasks, being excluded from special programs (e.g., programs for advanced learners), ethnic-racial teasing, or being provided curricula that fail to reflect diverse cultural practices (Douglass et al., 2016). The disproportionate use of punitive discipline strategies with students of color is another example of racial discrimination at an institutional level (Skiba et al., 2014). So, too, are policies permitting racially charged language under the guise of “free speech.” Notably, the impacts of racial discrimination can be similar to that of ACEs and acute traumatic events (Schulz & Rubel, 2011).

According to research, Black students face the highest rates of disciplinary action in both public and private schools, even though research indicates they are no more likely to misbehave than white students (J. Peterson et al., 2021). During the 2013–2014 school year, Black students were four times more likely to receive an out-of-school suspension than their white counterparts (J. Peterson et al., 2021). A 2017–2018 study found that Black students continue to be the most over-disciplined group across all categories of punitive discipline in schools, including suspension, expulsion, arrest, restraints, referral to law enforcement, and transfers to alternative schools for disciplinary reasons (Epstein et al., 2020). Importantly, this study reveals that girls of color were disciplined at higher rates than their white peers, often to a greater extent than the disparities observed between boys of color and white boys (Epstein et al., 2020). These outcomes reflect a persistent and systemic pattern of inequity and raise concerns about the long-term consequences of exclusionary disciplinary practices in schools.

## 3. Childhood Trauma

Exposure to ACEs is often conflated with trauma exposure, but they are not necessarily the same. As noted above, some ACEs are indeed traumatic (e.g., being abused, witnessing others being violently harmed) and their effects manifest similarly. However, trauma is the emotional response to an adverse event rather than the event itself (Jaycox et al., 2009). Trauma can be caused by exposure to single events (acute trauma) or by repeated events that last over days, weeks, months, or years, as is the case with racism, discrimination, and repeated acts of harassment and bullying experienced by some children (e.g., those with minoritized social identities and disabilities) (Herrenkohl et al., 2021). Physical, emotional, and sexual abuse; neglect; bullying; and the effects of long-term illness can all contribute to chronic trauma reactions that include depression, anxiety, and low self-esteem in children, adolescents, and adults (Jae, 2024; see additional information in the section below on Post-Traumatic Stress Disorder).

Complex trauma describes “both children’s exposure to multiple traumatic events—often of an invasive, interpersonal nature—and the wide-ranging, long-term effects of this exposure” (Spinazzola et al., 2018). Complex trauma tends to occur early in life and disrupts many aspects of a child’s development, including their formation of self and ability to form attachments (Griffin, 2018). Importantly, for racialized and minoritized students, experiences of microaggressions (everyday acts of racism) as well as interpersonal, institutional, and systemic racism contribute to a cumulative burden that can appear as complex trauma (Cénat et al., 2022). Reference to “complex racial trauma” underscores the collective, historical, and intergenerational experiences of racism that are perpetrated against children of color in Western society (Cénat et al., 2022).

## 4. Post-Traumatic Stress Disorder

Without appropriate intervention and support, traumatic stress symptoms can escalate into post-traumatic stress disorder (PTSD), a psychiatric condition that may develop after experiencing or witnessing traumatic events (American Psychiatric Association, 2022). PTSD can involve symptoms such as intrusive memories, heightened anxiety, and ongoing distress that interferes with daily functioning. According to the National Center for PTSD, among children and teens who have experienced a traumatic event, up to 15% of girls and 6% of boys develop PTSD (PTSD: National Center for PTSD, n.d.). Risk factors for developing PTSD include the severity of the trauma exposure, a child’s proximity to the traumatic event(s), and other factors (PTSD: National Center for PTSD, n.d.).

Children who develop PTSD are especially susceptible to adverse mental health and physical health conditions later in life, such as cardiovascular disease, diabetes, chronic pain, cancer, and autoimmune disorders (Wachen et al., 2019). PTSD negatively affects physical health outcomes through several pathways, including engagement in health risk behaviors (e.g., smoking or substance use) and co-occurring depression (Wachen et al., 2019). Early intervention is critical to preventing the long-term physical health consequences of childhood trauma.

## 5. Trauma and Child Development

Exposure to traumatic events could potentially disrupt a child’s neurological and emotional development by repeatedly activating the body’s stress response system (S. Peterson, 2018). When faced with a threat, a child’s brain activates its fear response and shuts down all non-essential systems (Cross et al., 2017). Typically, once the perceived threat has passed, the parasympathetic nervous system will restart, reducing a child’s stress response and allowing the body to return to its normal state. However, if the stress response is triggered repeatedly over time, it may result in alterations to brain structure and function (Cross et al., 2017). In particular, trauma can affect a child’s prefrontal cortex, which governs executive functioning. Executive functioning processes enable planning, focusing, and multitasking, and disruptions can impair working memory, cognitive flexibility, and abstract thinking (Cross et al., 2017). Consequently, the effects of trauma on a child’s brain can translate into learning difficulties which frequently require targeted support in the academic setting (S. Peterson, 2018).

Trauma can also impair children’s emotion regulation and processing abilities by altering the development of the amygdala, leading many traumatized children to struggle to identify, express, or manage their emotions (S. Peterson, 2018). A child who has experienced trauma may have unpredictable or explosive emotional responses, whereas others can appear quiet or guarded (S. Peterson, 2018). Many children who experience trauma can have difficulty calming down when upset and experience feelings of fear, depression, and anxiety (S. Peterson, 2018). Ultimately, this emotional dysregulation can impede a child’s ability to form relationships and develop a healthy sense of self-worth and value (S. Peterson, 2018).

Additionally, these neurological impacts can affect a child’s behavior in the classroom and limit their ability to succeed academically. For instance, symptoms of trauma such as intrusive thoughts, irritability, or dysregulation can interrupt a child’s learning process (S. Peterson, 2018). Behavioral manifestations of trauma, including aggression or withdrawal, tend to make children more vulnerable to disciplinary action (S. Peterson, 2018).

## 6. Trauma and Student Learning

Physical manifestations are one of many indicators of children’s responses to trauma. This recognition underscores the critical need for schools to identify and address traumatic stress responses that may impede learning (Perfect et al., 2016). Without recognition and intervention, symptoms such as intrusive thoughts, irritability, and anxiety can interfere with the ability to concentrate, which is fundamental to acquiring and retaining new knowledge and information.

Other traumatic stress symptoms such as dysregulation, aggression against self and others, dissociation, and somatization present additional barriers to social–emotional and behavioral functioning as well as academic performance. Such symptoms pose a significant challenge for educators and highlight the profound impact trauma can have on a child’s learning and behavior, possibly leading to “deficits in development” (S. Peterson, 2018).

The disruptions to executive functioning and social–emotional development erect barriers within the school setting that are often compounded by the far-reaching effects of chronic trauma, such as domestic violence, community violence, and homelessness. All of these contributors may cause a child to disguise their distress with aggressive or challenging behavior, which makes addressing the trauma difficult for school professionals (Miller, 2025). And while educators cannot single-handedly change the systems that use punitive measures for challenging behaviors, they can be intentional about prioritizing relationships, which, for students who have trauma histories or are experiencing trauma, is another obstacle to learning.

## 7. Schools as Re-Traumatizing Environments

Although schools provide a positive environment for many children, those with histories of trauma can experience them as unsafe or unwelcoming, particularly if they are subject to harsh treatment or harassment by their peers. Students who enter school with trauma histories may be re-traumatized by their experiences, which can exacerbate their symptoms (Koslouski et al., 2023). In 2021, more than one in five adolescents reported being bullied while at school (Irwin et al., 2021). In addition, students can experience trauma from school-based policies and practices that cause them harm (Dutil, 2020). As noted above, school policies related to punitive discipline have been shown to marginalize students of color, and to a lesser extent, students of lower socioeconomic status and students with disabilities (Dutil, 2020). National data from the Youth Risk Behavior Survey indicate that racial mistreatment in schools is common among students of color; in 2023, 20.4% of Black students, 13.8% of Hispanic students, and 13.5% of multiracial students reported being treated badly or unfairly at school because of their race or ethnicity (McKinnon et al., 2024). Further, at the systemic level, inequitable school funding contributes to poor working conditions and high staff turnover in certain schools, building a culture of distrust among staff and the families and adding to the burden of individual students (Fall & Billingsley, 2011; I. Morgan & Amerikaner, 2018).

Zero-tolerance school discipline policies, which have been widely implemented in schools since the 1990s, can lead to excessive suspensions, expulsions, and juvenile justice referrals (Dutil, 2020). Over the last decade, an increasing number of schools have begun reducing their use of punitive responses and instead adopting restorative practices (Evans & Gregory, 2020). However, during the 2021–2022 school year, a majority (62%) of U.S. public schools continued to utilize zero-tolerance policies or mandatory exclusionary penalties, reflecting the persistence of disciplinary structures alongside reform-oriented approaches (Perera & Diliberti, 2023). Policies and practices that remove students from school for disciplinary reasons are associated with increased risk for contact with the juvenile justice system (Skiba et al., 2014). Researchers use the term “school-to-prison-pipeline” to describe the disciplinary policies and practices that decrease the probability of school success for children and youth and instead increase their risk for crime and incarceration (Skiba et al., 2014). Higher levels of exclusionary discipline may heighten the risk of incarceration by increasing interactions with law enforcement and fostering academic disengagement that can lead to school dropout (itself a risk factor for delinquency and crime) (H. Morgan, 2021). In fact, one 2007 study found that being suspended increased the likelihood of school dropout by nearly 77.5% (Skiba et al., 2014). Students who stayed in school for a year after suspension experienced an average GPA decline of 0.88 points (Skiba et al., 2014). Ultimately, harsh disciplinary practices that target and criminalize Black students have been identified as one factor associated with the mass incarceration of Black individuals in the United States—a topic of major concern to advocates and some policy makers (H. Morgan, 2021).

## 8. Restorative Approaches

Restorative initiatives, encompassing both restorative practices (RPs) and restorative justice in education (RJE), are designed to enhance school culture by fostering stronger relationships. In this context, RJE serves as the philosophical foundation, while RP operates as its practical application. Restorative practices adopt a relationship-centered approach across the school community, addressing conflict through the repair of harm rather than punitive responses (Darling-Hammond, 2022). These practices present a credible alternative to punitive and exclusionary disciplinary strategies. Additionally, restorative approaches prioritize proactive measures that promote community building, effective communication, and conflict resolution skills.

A salient example of community-building in restorative practice is the implementation of circles—structured dialogues designed to facilitate sharing and collaborative problem-solving. Circles cultivate an atmosphere in which participants are encouraged to communicate openly and engage in active, respectful listening (Boyes-Watson & Pranis, 2015). Circles can focus on two principal considerations: addressing the needs of those harmed and of those responsible for causing harm and general community-building. Problem-solving circles routinely incorporate structured, reflective questions, such as: “What occurred?”, “Who has been affected by these actions?”, and “What steps are necessary to repair the harm?” Both proactive and responsive approaches to restorative practice are relationship-centered alternatives to traditional school discipline. The essential tenets of RP are rooted in indigenous communities and religious traditions where the concept of justice assumes that everyone in the community is connected (Evans & Gregory, 2020; Zehr, 2015). Therefore, when an injustice has occurred, it represents “a wound in the community, a tear in the web of relationships” (Zehr, 2015, p. 29).

Restorative practices are increasingly recognized as an important component of a nurturing, supportive, and safe learning environment, especially for students with trauma histories. A study conducted by the Brown Center on Educational Policy and the Rand Organization (Perera & Diliberti, 2023) suggests that while many schools are seeking alternatives to exclusionary and punitive approaches, many schools still allow teachers’ use of suspension and other punitive measures for low-level, non-violent student behaviors (Perera & Diliberti, 2023). Due to evidence that punitive discipline may heighten challenging behaviors and erode safe and nurturing learning environments, the last decade has witnessed schools beginning a shift from exclusionary, zero-tolerance, and aggressive punitive practices to more restorative initiatives (Evans & Gregory, 2020, p. 6).

The National Education Policy Center defined restorative justice education, which is a subset of restorative practice, as a comprehensive approach to shifting school culture and prioritizing relational pedagogies, justice and equity, resilience-building, and well-being. Building on the work of Paulo Freire and bell hooks, educators in RJE schools strive to ensure that the “vulnerable are cared for, the marginalized are included, the dignity and humanity of each person in the educational setting matters, and everyone’s needs are heard and met” (Evans & Gregory, 2020, p. 3).

Research suggests that restorative initiatives may have the potential, if not fully realized, to reduce disparities in suspensions (Evans & Gregory, 2020). For example, the utilization of restorative practices in some large urban districts shows potential. In Oakland and Los Angeles, California, where school districts are adopting comprehensive discipline reforms and restorative practices, the suspension gap between Black and White students has decreased (Evans & Gregory, 2020). Furthermore, replacing exclusionary practices with restorative justice initiatives could potentially reduce the high rates of disciplinary action experienced by Black students. For instance, when six elementary and middle schools in Texas piloted restorative discipline programs in an attempt to decrease their suspension rates, they experienced a 70% reduction in in-school suspensions and a 77% reduction in out-of-school suspensions (H. Morgan, 2021).

Additionally, a comprehensive restorative approach involves more than a response to challenging behavior; it proposes to “transform schools from rule-based institutions to relationship-based communities” (Evans & Gregory, 2020). Such a transformation involves enacting several key principles: replacing punitive models of discipline with restorative models that promote repair of harm; moving from systems of social control to systems of social engagement; confronting hierarchical and authoritarian systems that instill attitudes of obedience and conformity; and making a commitment to disrupting oppressive structures and systems (Evans & Gregory, 2020).

This relational shift is further supported by Trauma-Informed Programs and Practices (TIPPS), an evidence-based framework that integrates trauma-informed principles with restorative practices to guide implementation in classrooms, schools, and school systems. Unlike restorative practices, which broadly focus on repairing harm and building community, TIPPS translates research into practice and shares strategies that guide district leaders and school professionals in creating safe, nurturing, inclusive environments for all students, thereby helping students realize their potential and become more resilient to the effects of trauma. Building and sustaining relationships are central to the TIPPS framework. TIPPS emphasizes that strong, positive student–teacher relationships are an essential component of prosocial classroom environments. Trusting relationships are particularly important for students who have experienced adversity and trauma, as they can support the development of emotional regulation and adaptive coping skills (TIPPS, 2022).

Maintaining order through compliance and demonstrating discipline through control has been the cornerstone of classroom management for generations. While some of these practices are intended to provide structure, they can send a message of rejection, shame, and disconnection for all students, especially those with trauma histories (Perera & Diliberti, 2023). Restorative practices reimagine the relationships between students and teachers and prioritize healing rather than punishment as the consequence. Punitive and exclusionary measures can heighten the negative behaviors while restorative practices shift discipline away from harsh and restrictive responses toward relationship-building approaches that are more likely to foster safe, equitable, and inclusive learning environments. With a restorative approach, discipline is no longer about exclusion; it is about inclusion and connection.

Key principles of RJE include transforming schools from rule-based institutions to relationship-based communities; replacing punitive models of discipline with restorative models that promote repair of harm; and moving from systems of social control to systems of social engagement (Evans & Gregory, 2020, p. 23). Adhering to these principles has the potential to reduce suspension rates and narrow the disparities in discipline between Black and white students (Evans & Gregory, 2020, pp. 9–10). Furthermore, students have reported benefits from restorative processes such as circles and conferences, including strengthened social and emotional skills (Evans & Gregory, 2020, p. 11). As the TIPPS framework suggests, “When students feel that school professionals not only care about them as learners but also as people with lives outside of school, they are more likely to feel emotionally and physically safe, supported, and appreciated” (TIPPS, 2022).

To illustrate how punitive and restorative approaches can lead to very different outcomes, we begin with the case of Travis (found in our TIPPS Casebook) and an example of a punitive response to student behavior (TIPPS, 2024):

*Travis is a high needs middle school student, who has been living with his grandparents since his mother was incarcerated last Spring. He is having trouble getting started on a family tree research project and asks to go to the bathroom but doesn’t return in a timely manner. Upon his return from the bathroom, his teacher, Mr. Sanderson, tells him he missed some of the instructions and prompts him to begin filling out the template. Travis tells him he doesn’t know any of the information on the template, so the teacher suggests he fill in what he can and ask his parents for help after school.*

*Travis reminds Mr. Sanderson that he lives with his grandparents and that they don’t have time to help him with homework. Mr. Sanderson, a seasoned teacher with a reputation for having a rigorous curriculum and a no-nonsense approach to classroom management, offers no response but continues to point to the assignment and suggest that he gets started. Travis sits for a few minutes, shaking his legs and humming softly to himself. Frustrated, Mr. Sanderson demands that Travis start the assignment. Instead of starting the assignment, Travis pulls out his phone, puts in his earbuds, and begins listening to music. Mr. Sanderson raises his voice and demands that Travis put his phone away and start the assignment. Without warning, Travis stands up, yells expletives at the teacher, and knocks over his desk. He continues to yell obscenities and throw his papers off his desk until a classmate calms him down. Mr. Sanderson phones the office and tells them that he needs Travis to be removed from his classroom and that he will submit a violation report by the end of the hour. Travis is physically removed from the classroom by two administrators as his classmates could hear him yelling down the hall.*

## 9. Trauma-Informed Approaches in Schools: TIPPS

A trauma-informed approach can help schools better serve students who have experienced trauma by realizing, recognizing, responding, and resisting re-traumatization in the school setting (Griffin, 2018). As mentioned previously, TIPPS is a system-focused framework that can help schools create safe, nurturing, and inclusive learning environments for all students (TIPPS, 2022). Though distinct from RJE, the TIPPS framework incorporates key restorative practices, including developing relationships that protect against the harmful effects of systemic inequities and punitive disciplinary approaches in the school environment. Through core pillars, such as TIPP 3: Increase Awareness of Biases and Stereotypes; TIPP 5: Develop and Model Positive Relationships; and TIPP 6: Reduce Punitive Discipline, the TIPPS framework guides schools in implementing equitable, supportive practices that foster students’ sense of value, belonging, and connection.

One of the first steps to creating a school-wide approach to trauma is reducing and preventing experiences that may re-traumatize children (Herrenkohl et al., 2020). The TIPPS framework seeks to prevent re-traumatization by ensuring all staff are aware of the effects of punitive discipline and equipped with strategies to positively respond to student behaviors. This can help shift the culture and climate of schools to be more nurturing and conducive to learning (Herrenkohl et al., 2019). The above scenario highlights the connection between students’ trauma histories and the manifestation of challenging classroom behaviors. A proactive approach to restorative practice suggests that much of the conflict and behavioral challenge in this scenario could have been prevented if Mr. Sanderson had built a positive relationship with Travis before the conflict arose. Having some background knowledge about Travis’ living situations would help Mr. Sanderson understand that focusing on family might trigger an intense reaction. Being aware of students’ lives outside the classroom and being mindful of their experiences helps teachers develop and model positive relationships and offer restorative responses to classroom conflict.

In this example, the teacher prioritized content delivery over the student’s relational and emotional needs. Viewed through a trauma-informed, restorative lens, this scene illustrates that how teachers relate to their students is as important as how effectively they teach about an academic subject or topic (TIPPS, 2022). A restorative response to this scenario would focus on repairing harm and rebuilding trust. Unlike exclusionary disciplinary practices, restorative practices seek to address the root causes of challenging student behavior while enhancing positive school culture and academic engagement (Darling-Hammond, 2022).

If Mr. Sanderson had shaped his classroom culture with restorative, trauma-informed principles in place, the interaction might have looked much different. A proactive restorative interaction:

*Teacher: (Notices the students’ body language) Travis, what’s up? Can I help you get started on this assignment? I know you like to draw, maybe some of your responses can be illustrated.*

*Student: I don’t want to do it.*

*Teacher: Okay. I hear you. It seems like a lot of work. We can break it into smaller chunks.*

*Student: I don’t want to. I’m not gonna do it.*

*Teacher: Is there something about the assignment that is bugging you? Maybe we can change part of it?*

*Student: I don’t wanna talk about my family.*

*Teacher: Thank you for letting me know. Can we work together on changing up some of the prompts so we can hit the benchmarks of the assignment?*

*Student: I’ll think about it.*

*Teacher: Cool! I’ll be back in a few minutes to check in*

*Student: Ok. (Asks to go to the bathroom)*

*Teacher: Sure. Let’s work together when you come back from the restroom.*

If the disruptive encounter did take place, Mr. Sanderson could still rely on a restorative approach to respond to the challenging behavior; physical removal and other exclusionary practices do not have to be the only options. A responsive restorative interaction (Collaborative for Academic, Social, and Emotional Learning (CASEL), 2025):

*(Student has knocked over his desk and yelled expletives)*

*Teacher: Travis, I can see that you are very upset. I want to help but need us to be safe.*

*Student: (Pacing)*

*Teacher: Let’s take a minute to breathe (Teacher breathes)*

*Student: (Breathes)*

*Teacher: (Privately and quietly) What happened?*

*Student: I got pissed and knocked over my desk*

*Teacher: What were you thinking at the time?*

*Student: I didn’t want to do it*

*Teacher: Ok. I hear you. What’s up?*

*Student: I’m pissed and feel stupid. I don’t want to do stuff about my family.*

*Teacher: Travis, it sounds like you were frustrated because the assignment was uncomfortable. I’m sorry for that, but it’s not okay to throw things in class.*

*Student: Yea.*

*Teacher: So, who do you think your actions affected?*

*Student: Probably the class and you*

*Teacher: What can you do to make things right?*

*Student: Apologize and get to work*

*Teacher: Thank you for talking with me, Travis. I will take a look at the assignment and make some adjustments. Fair? (starts to pick up the papers)*

*Student: Fair. (Begins to pick up papers and desk) I got this. Can I go to the bathroom?*

*Teacher: Of course. Let’s work together when you come back from the bathroom.*

Shifting school culture from a punitive to a trauma-informed restorative approach requires a commitment to both practice and policy. Research suggests that practices, rather than programs alone, are the determinants of outcomes (Darling-Hammond, 2023). In other words, consistently enacting restorative practices, rather than simply adopting a program or framework, may be more likely to support meaningful change. Implementing prosocial and restorative practices (both proactive and responsive) can strengthen relationships, increase equity, and prioritize a sense of safety and belonging (Darling-Hammond, 2022). Darling-Hammond (2022) recommends the following:

- Prioritize relationships and replace punitive discipline practices, including zero tolerance policies
- Include data on exclusionary practices (suspension and expulsion), particularly focused on reducing disparities among students of color and students with disabilities; restorative practices, particularly centered on the use of restorative strategies (i.e., community building circles, restorative conversations, and conflict resolution); and a school climate that measures student and staff perception of safety
- Integrate this data into state/local district ratings, using it to guide school improvement plans
- Increase staff investment by providing long-term, engaging and relevant professional learning
- Strengthen connections within the school community by centering and representing student voices from across all social segments

## 10. Discussion

Punitive disciplinary practices can further marginalize students and contribute to disengagement from school (Dutil, 2020; H. Morgan, 2021). Instead, the use of restorative practices to address misbehavior can help establish a school environment where students feel supported and nurtured. Despite these benefits, implementing restorative practices in schools can seem like a daunting task, as it often requires shifts in school culture, staff training, and a rethinking of traditional, well-established punitive discipline practices. Teacher skepticism toward restorative practices is both common and understandable, especially when educators are concerned that restorative approaches may be too time-consuming or may not adequately address serious behavioral concerns. However, when embedded within a broader trauma-informed, school-wide framework like TIPPS, restorative practices are no longer alternatives to suspension or isolated classroom strategies; rather, these practices are part of a coordinated approach to building safe, inclusive, and nurturing learning environments through relationships and accountability.

The following narrative illustrates how an educator’s initial resistance to restorative practices can change when these approaches are implemented consistently, supported by school leadership, and connected to a systemic effort to transform school culture. In “Empathy, Equity, Empowerment: Using Restorative Practices to Build Character and Community While Reducing Suspensions,” Christopher Martin, a middle school science teacher, describes his initial skepticism toward restorative practices and the school-wide implementation process that eventually shifted his perspective (Martin, 2015). Martin originally worked in a school in Denver, Colorado, that was built around a punitive model of behavior management. School staff would record merits and demerits in a computer system that tracked and shared this information with administrators, students, and families. For some students, this system was effective. Many other students, however, were unable to meet expectations and ended up being pushed out of the school. Students who struggled with academics or impulse control were viewed negatively and intentionally excluded, Martin reflects. Instead of fostering a sense of community and trust, the school was acting out of fear and compliance.

A few years later, Martin started teaching at a school in Denver that served a predominantly Latine population. After initially struggling to maintain a student body and positive reputation, the administrators worked tirelessly to make their school a place where the community felt proud to send their children. Martin notes that one of the school’s greatest agents of change in achieving a reputable status was the implementation of restorative practices. Between 2007 and 2014, enrollment doubled from 300 to 600 students, and at the same time, incidents of out-of-school suspension dropped from nearly 300 to about 50 per year.

Initially, Martin felt both intimidated by and skeptical of his school’s use of restorative practices. Teachers were encouraged to use restorative responses to behavior in their classrooms, but Martin ignored the guidance and continued to send his students to the discipline office. Though he was impressed by the guidance counselor’s ability to guide his students through restorative conversations, he remained skeptical. Martin describes his frustration with the process; when he did try to have a restorative conversation with a student, he felt angry when they returned to the classroom and continued to make the same behavior choices.

Meanwhile, the school continued to receive positive feedback about their intentional work to foster a trusting community. Over the summer, a group of instructors deeply committed to sustaining and strengthening the school’s restorative practices met weekly to evaluate which policies were effective and which needed refinement. Martin decided to attend the weekly meetings and started to realize how and why the school’s restorative approach worked. Upon reflection, Martin realized that removing students from the classroom might contribute to feelings of isolation, fear, and shame, rather than improved behaviors.

Educators were trained to implement restorative practices in each of their classrooms. The school supported fidelity by using a comprehensive intervention manual, a product of the staff summer meeting sessions. If teachers encountered a behavior in need of redirection, they were instructed to first use a warm correction. If the behavior continued or reached the point of needing a removal, students were asked to take a break and complete a refocus form that consisted of five questions: what happened, who was affected, what are you able to take responsibility for, what could you have done differently in this situation, and what are you willing to do to make things right. Afterwards, the teacher and the student would share a quick conversation to assess the student’s empathetic reflection and readiness to rejoin. The refocus does not guarantee that mistakes will not happen again, but it does ensure that the student has maintained their dignity, has been held accountable, and better understands how this behavior can be avoided in the future. While the whole process was intended to take about ten minutes, Martin admitted that restorative practices are not a quick fix; building trust and fostering connection takes time and practice. However, with effective support from school administrators, schools can build a multi-tiered system of support that changes student lives.

As Martin suggests, shifting from punitive disciplinary measures to restorative approaches can seem challenging, time-consuming, and counterproductive; critics of restorative practices fear that they could undermine the role of punitive consequences, resulting in a permissive learning environment (Goold, 2023). In Martin’s case, his perspective shifted when restorative practices were incorporated as part of a school-wide intervention supported by routine procedures, staff collaboration, and administrative leadership, rather being treated as an isolated strategy dependent on individual teacher implementation. This aligns with the TIPPS framework, which encourages system-level change by guiding efforts to transform schools into safe, nurturing, and inclusive learning environments that strengthen relationships and provide opportunities for all students and staff to develop resilience and positive coping skills (TIPPS, 2022).

Martin’s experience speaks to practical steps for supporting effective implementation of restorative or trauma-informed practices. First, school administrators should acknowledge educators’ concerns about restorative practices, rather than dismiss them. Opportunities for professional development can provide a space for discussion and additional learning. Second, consistent with the TIPPS framework, implementation should be schoolwide. Not only do individualized approaches place unnecessary stress on teachers, but they can also stigmatize students who need help (TIPPS, 2022). Implementing a comprehensive, trauma-informed approach in schools can lead to the development of an equitable environment that benefits students and teachers equally (TIPPS, 2022).

## 11. Future Directions

Schools can begin incorporating restorative practices by holding professional development to teach staff about the adverse effects of punitive discipline and equip them with the necessary tools to implement restorative practices. The TIPPS workshop series and online modules provide an overview of these topics and equip school professionals with trauma-informed practices in 6–10 h. Additionally, educators can consult the TIPPS Tools and Resources page to support the implementation of trauma-informed and restorative practices with fidelity in school settings. With training and resources, school and district leaders can integrate restorative practices into strategic, school-wide goals (Goold, 2023). For example, a school may set goals such as “conduct a restorative circle every week,” which provides teachers with flexibility and autonomy in achieving this goal (Dhaliwal et al., 2025). This also helps sustain restorative approaches, so they remain at the forefront after training.

Implementing restorative practices can feel overwhelming for schools balancing competing priorities. Thus, it is important that there is alignment between restorative goals and teacher readiness to change for successful implementation (Vincent et al., 2021). While there may be staff buy-in and professional development around the topic, many educators can feel unprepared to facilitate restorative practices (Moran et al., 2024). Given this challenge, additional coaching with an experienced facilitator may help improve staff members’ confidence in trying out these strategies (Parker & Bickmore, 2020). Further, ongoing professional development is needed to continue building restorative skills and to account for staff turnover (Evans & Gregory, 2020). Shifting to a restorative approach is not easy; however, these approaches can build students’ prosocial skills and reduce problematic behaviors, which can pay dividends in the long term (Evans & Gregory, 2020).

Moving from punitive discipline to restorative practices requires a broader cultural and systemic change in how schools understand behavior, accountability, and student support. Although implementation can be complex and time-intensive, restorative practices are effective in the long run. A study of 485 middle schools across multiple U.S. districts found that exposure to restorative practices was associated with improved academic achievement, reduced suspension rates, narrower racial disparities in discipline and achievement, and improvements in student behavior and school safety (Darling-Hammond, 2022). Unlike the well-established negative effects associated with exclusionary punishments (Skiba et al., 2014), restorative practices can improve school climate, encourage academic success, and foster student well-being. Ultimately, effectively supporting students who have experienced trauma and preventing re-traumatization requires schools to make sustained, intentional commitments to relationship-building, accountability, and open communication.

Future research should focus on examining restorative practices within larger, schoolwide efforts to address issues of trauma and promote equitable school climates. Rather than studying restorative practices as isolated interventions, researchers should take a systems-wide approach and consider how schools adopt, implement, and sustain them across diverse contexts. Qualitative, quantitative, and mixed-methods studies will be essential to what factors support or prevent meaningful implementation while also highlighting multiple perspectives, including those of students, educators, and administrators. This work aligns with ongoing efforts to develop and study structured implementation support for our TIPPS framework in various settings, such as rural communities. Together, these efforts will contribute to the growing body of research on restorative frameworks and support schools in developing safe, inclusive environments that both mitigate the effects of trauma and enhance student engagement and achievement.

## 12. Conclusions

Trauma, an emotional response to an unexpected, life-threatening situation, has lasting and adverse effects on a child’s behavior and physical, social, or emotional well-being (Jaycox et al., 2009). Specifically, trauma can impact a child’s neurological development, leading to lasting changes in brain structure and function (Cross et al., 2017). Changes to the prefrontal cortex can lead to impaired executive functioning (Cross et al., 2017), while changes in the amygdala may cause emotional dysregulation or heightened fear responses (S. Peterson, 2018). These neurological impacts might disrupt children’s ability to learn and regulate their behavior, as trauma-related symptoms such as intrusive thoughts, irritability, aggression, or withdrawal both hinder academic success and increase the likelihood of disciplinary action (S. Peterson, 2018). Importantly, systemic and interpersonal racism in schools and society is associated with increased exposure to adverse childhood experiences among minoritized youth (Dutil, 2020; Swedo et al., 2024). This could contribute to greater vulnerability to the effects of trauma on learning and student well-being and, ultimately, persistent racial disparities in academic achievement and physical and mental health outcomes (Swedo et al., 2024; Darling-Hammond & Ho, 2024).

The implementation of zero-tolerance policies in schools during the 1990s fostered an overreliance on punitive discipline, perpetuating the school-to-prison pipeline (Mallett, 2015). Research suggests that Black students are suspended, expelled, and arrested at much higher rates than their white peers, leading to disproportionate contact with the juvenile justice system (Darling-Hammond & Ho, 2024). Exclusionary discipline practices can erode self-esteem, heighten feelings of rejection, trigger aggressive behaviors, and ultimately, maintain a cycle of trauma and racial inequity (Skiba et al., 2014). Restorative practices, on the other hand, are an essential tool for building a nurturing, supportive, and safe learning environment, especially for students with trauma histories. To protect the well-being of children and advance racial equity, it is imperative that schools implement trauma-informed approaches that correspond with the TIPPS framework while also embedding restorative values and principles into their practices.

## Data Availability

No new data were created or analyzed in this study.
